# Carcinoma ex Pleomorphic Adenoma With an Epithelial‐Myoepithelial Carcinoma Component in the Submandibular Gland: A Case Report

**DOI:** 10.1155/crid/7698933

**Published:** 2026-07-17

**Authors:** Tomohiro Yasuhara, Masanori Masui, Yuki Kunisada, Hiroaki Takakura, Eiji Iwata, Koichi Kadoya, Koki Umemori, Norie Yoshioka, Keisuke Nakano, Soichiro Ibaragi

**Affiliations:** ^1^ Department of Oral and Maxillofacial Surgery, Faculty of Medicine, Dentistry and Pharmaceutical Sciences, Okayama University, Okayama, Japan, okayama-u.ac.jp; ^2^ Department of Oral Pathology and Medicine, Faculty of Medicine, Dentistry and Pharmaceutical Sciences, Okayama University, Okayama, Japan, okayama-u.ac.jp

**Keywords:** carcinoma ex pleomorphic adenoma, case report, epithelial-myoepithelial carcinoma, salivary gland neoplasm, submandibular gland

## Abstract

**Background:**

Carcinoma ex pleomorphic adenoma (CXPA) is an uncommon malignant salivary gland tumor arising from pleomorphic adenoma, whereas epithelial‐myoepithelial carcinoma (EMC) is a rare low‐grade salivary gland malignancy. CXPA with an EMC component in the submandibular gland is uncommon.

**Case Report:**

A 65‐year‐old man presented with a painless, firm mass in the left submandibular region and weakness of the left lower lip. Computed tomography and magnetic resonance imaging demonstrated an irregular submandibular mass, and FDG‐PET/CT showed increased uptake (maximum standardized uptake value, 9.2). Core biopsy suggested pleomorphic adenoma; however, the clinical and radiological findings strongly indicated malignancy. Incisional biopsy was therefore performed under general anesthesia, with a plan to proceed to definitive treatment if malignancy was confirmed. Intraoperative histopathological examination revealed a malignant salivary gland tumor, and tumor resection with neck dissection was completed during the same procedure. Histopathological examination showed invasive CXPA with an EMC component, accompanied by extracapsular invasion exceeding 6 mm, perineural invasion, and extension into adjacent muscle (pT3N0). Although surgical margins were negative, pulmonary metastases developed 12 months after surgery, followed by suspected pleural dissemination and spinal canal extension. The patient declined further aggressive treatment and died of the disease 48 months after surgery.

**Conclusion:**

This case highlights the importance of integrating clinical, radiological, and pathological findings for accurate diagnosis of salivary gland tumors, particularly when biopsy results are inconsistent with clinical suspicion.

## 1. Introduction

Carcinoma ex pleomorphic adenoma (CXPA) represents malignant transformation of a pre‐existing pleomorphic adenoma (PA) and is clinically important because residual benign and malignant components may coexist within the same lesion, making diagnosis difficult in limited biopsy specimens [1, 2]. Although the parotid gland is the most frequent site, CXPA can also arise in the submandibular gland [2]. Most malignant salivary gland tumors show ductal epithelial differentiation, whereas tumors with prominent myoepithelial differentiation are uncommon [3].

Epithelial‐myoepithelial carcinoma (EMC) is an uncommon salivary gland carcinoma characterized by biphasic epithelial and myoepithelial differentiation [4–6]. When EMC occurs as the carcinomatous component of CXPA, particularly in the submandibular gland, preoperative diagnosis may be challenging because limited biopsy specimens may sample only the benign PA component or show biphasic/basaloid features that overlap with PA. Herein, we report an uncommon case of CXPA with an EMC component arising in the submandibular gland, highlighting the diagnostic challenges associated with discordant clinical, radiological, and pathological findings.

## 2. Case Report

A 65‐year‐old man was referred to our department in early March 2021 because of swelling in the left submandibular region. His medical history was significant for hypertension, and his family history was unremarkable. The swelling was first noticed by a family member, after which the patient was referred to our hospital for further evaluation and treatment.

**Figure 1:**
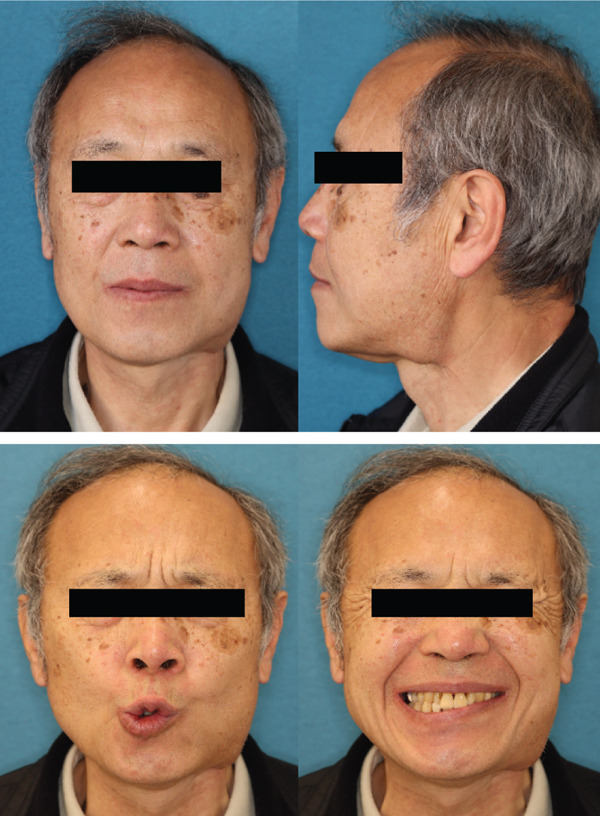
Clinical appearance at the initial presentation. A painless, firm mass approximately 30 mm in diameter was palpable in the left submandibular region and appeared adherent to the mandible. Mild weakness of the left lower lip was also observed.

On extraoral examination, a painless, firm mass measuring approximately 30 mm was palpable in the left submandibular region and appeared adherent to the mandible (Figure 1). Mild weakness of the left lower lip was present, suggesting possible involvement of the marginal mandibular branch of the facial nerve. No notable intraoral findings were observed.

**Figure 2:**
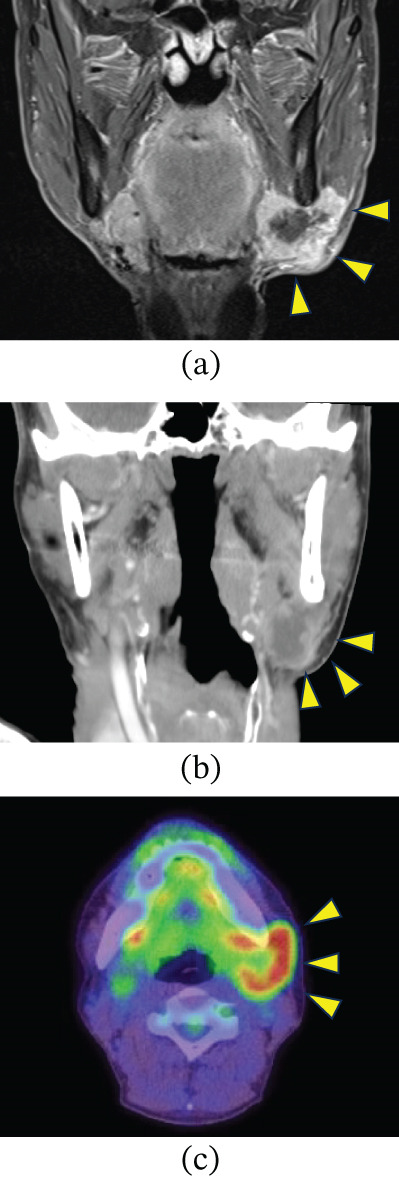
Radiological findings at the initial presentation. (a) Contrast‐enhanced CT showing a mass lesion with internal low‐attenuation areas in the left submandibular region, without mandibular destruction or resorption. (b) Contrast‐enhanced MRI showing an irregularly marginated mass replacing the left submandibular gland, with heterogeneous internal signal intensity and predominantly peripheral heterogeneous enhancement. (c) FDG‐PET/CT demonstrating increased uptake in the left submandibular mass (maximum standardized uptake value, 9.2), without evidence of another primary malignancy or distant metastasis.

Contrast‐enhanced computed tomography revealed a mass lesion in the left submandibular region with internal low‐attenuation areas and no evidence of mandibular destruction or resorption (Figure 2a). Contrast‐enhanced magnetic resonance imaging demonstrated enlargement of the left submandibular gland, which was replaced by an irregularly marginated mass. The lesion showed heterogeneous internal signal intensity, including fluid‐like areas and focal low signal intensity on T2‐weighted images, with predominantly peripheral heterogeneous enhancement (Figure 2b). Based on these clinical and imaging findings, the lesion was considered to be malignant, and 18F‐fluorodeoxyglucose positron emission tomography/computed tomography (FDG‐PET/CT) was performed. FDG‐PET/CT demonstrated increased uptake in the left submandibular mass, higher than that in the surrounding normal soft tissue, with a maximum standardized uptake value (SUVmax) of 9.2, and no evidence of another primary malignancy or distant metastasis (Figure 2c). Laboratory findings were unremarkable. The clinical diagnosis was a malignant tumor of the left submandibular gland.

**Figure 3:**
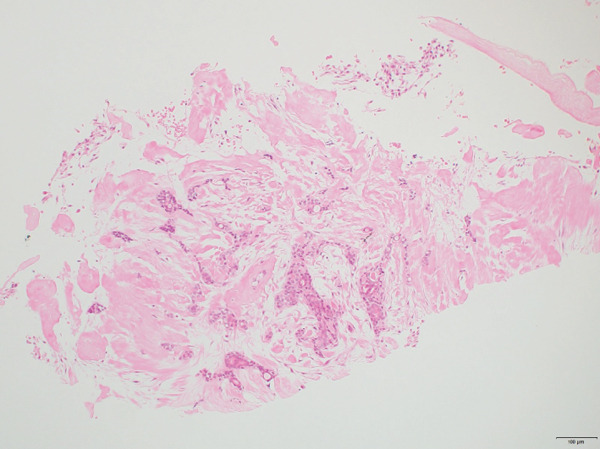
Histopathological findings of the core needle biopsy specimen. Bilayered duct‐like structures composed of ductal epithelial cells with eosinophilic cytoplasm and surrounding basaloid cells are observed. Tumor cells with duct‐like architecture are also seen proliferating diffusely within abundant stroma. No definite malignant component was identified in the sampled tissue, and the findings were interpreted as pleomorphic adenoma‐like, although the possibility of CXPA was noted (hematoxylin‐eosin stain, original magnification ×10).

A core needle biopsy using an 18‐gauge needle revealed bilayered duct‐like structures composed of ductal epithelial cells with eosinophilic cytoplasm and surrounding basaloid cells with round to oval nuclei. Tumor cells with duct‐like architecture also proliferated diffusely within abundant stroma. No definite malignant component was identified in the sampled tissue; therefore, the biopsy findings were interpreted as PA, although the possibility of CXPA was noted (Figure 3). Because this provisional pathological interpretation was discordant with the clinical and imaging findings, which strongly suggested malignancy, incisional biopsy under general anesthesia was planned, with a strategy to proceed directly to definitive resection if malignancy was confirmed intraoperatively.

**Figure 4:**
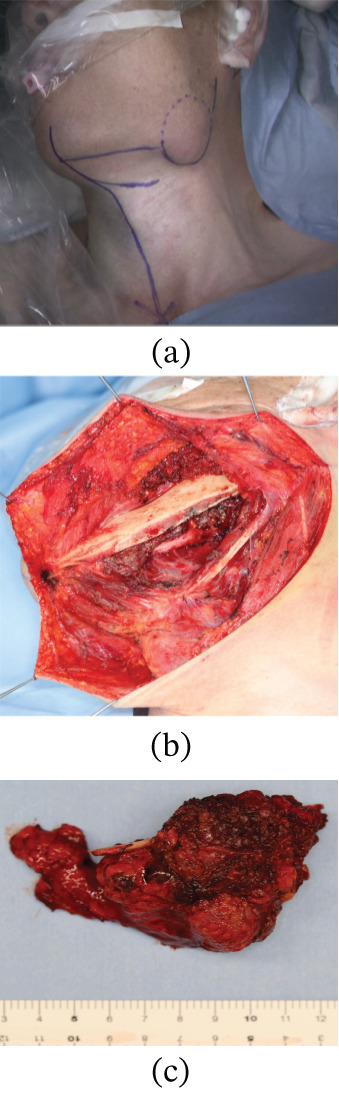
Intraoperative findings. (a) Skin incision design. (b) Operative field after tumor resection. En bloc resection of the left submandibular malignant tumor with selective Level I neck dissection was performed. Marginal mandibulectomy and resection of adjacent soft tissue, including parts of the masseter and medial pterygoid muscles, were performed to secure an adequate oncologic margin. (c) Resected specimen.

Intraoperative histopathological examination of the incisional biopsy specimen revealed a malignant salivary gland tumor. Consequently, en bloc resection of the left submandibular tumor, including marginal mandibulectomy, the platysma muscle, and parts of the masseter and medial pterygoid muscles, was performed during the same procedure (Figure 4). Although preoperative imaging showed no cortical bone destruction or bone marrow invasion of the mandible, the superior aspect of the tumor was in contact with the inferior border of the mandible. Therefore, marginal mandibulectomy was performed not for treatment of confirmed bone invasion, but to secure an adequate oncologic margin after malignancy had been confirmed by intraoperative histopathological examination.

Because lower‐lip weakness had been present preoperatively, involvement of the marginal mandibular branch of the facial nerve was suspected. During tumor resection, the platysma muscle adjacent to the tumor was included in the resection specimen to ensure an adequate oncologic margin. The marginal mandibular branch was not dissected separately for preservation in the vicinity of the tumor and was sacrificed en bloc with the surrounding soft tissue. Selective Level I neck dissection, including the submandibular and submental regions, was performed en bloc with the primary tumor, and Level II lymph nodes were also examined histopathologically.

**Figure 5:**
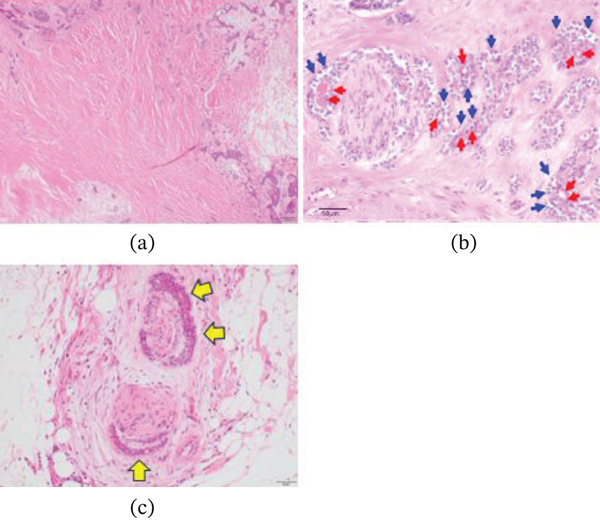
Histopathological findings of the resected specimen. (a) Pleomorphic adenoma component with marked stromal hyalinization. (b) Biphasic tumor cells composed of inner ductal epithelial cells and outer myoepithelial cells. Red arrows indicate inner ductal epithelial cells, and blue arrows indicate outer myoepithelial cells. (c) Biphasic glandular structures composed of cuboidal ductal epithelial‐like cells and basaloid cells with clear cytoplasm, with perineural invasion by tumor cells (arrows). Hematoxylin‐eosin stain; original magnification ×4 in A and ×20 in B and C.

Histopathological examination of the resected specimen showed biphasic proliferation of cuboidal ductal epithelial‐like cells and basaloid/myoepithelial cells arranged in duct‐like structures and trabecular nests. A PA component with marked stromal hyalinization was also identified (Figure 5a). Extracapsular invasion exceeded 6 mm, and perineural invasion and extension into adjacent muscle were present, corresponding to invasive CXPA with high‐risk pathological features (Figure 5b,c). Immunohistochemically, the ductal epithelial cells were positive for AE1/AE3 and epithelial membrane antigen, whereas the outer tumor cells were positive for p63, p40, and CK14 and showed partial positivity for smooth muscle actin. p53 immunostaining exhibited a wild‐type pattern, with scattered positive tumor cells. The Ki‐67 labeling index showed regional heterogeneity: approximately 3% in the intracapsular component, approximately 10% as an overall estimate, and 27% in hot spots of the extracapsular invasive component. Histopathological examination showed no lymph node metastasis in the Level I lymph nodes adjacent to the primary tumor (0/7) or in the Level II lymph nodes (0/2), and the nodal status was classified as pN0. The final pathological diagnosis was CXPA with an EMC component. The tumor was classified as pT3N0 because of extraparenchymal extension into adjacent muscle, despite a maximum diameter of approximately 30 mm.

Adjuvant radiotherapy was considered because of the presence of high‐risk pathological features, including perineural invasion and adjacent muscle involvement. However, because complete resection with negative margins had been achieved and the patient preferred to avoid treatment‐related toxicity after discussion of the risks and benefits, a policy of close observation was adopted. At 12 months after surgery, multiple pulmonary nodules were detected on follow‐up imaging. The lesions gradually increased in size over time and were considered consistent with pulmonary metastases. After multidisciplinary discussion, surgical management was selected, and partial resection of the right lung was performed at 20 months after the initial surgery, followed by partial resection of the left lung at 22 months. Histopathological examination confirmed that these lesions were metastases from the primary salivary gland carcinoma. Subsequently, additional pulmonary lesions were treated with radiofrequency ablation.

Despite these interventions, the disease gradually progressed, with enlargement of multiple pulmonary metastases. Additional immunohistochemical evaluation of the primary tumor was performed to explore potential systemic treatment options. Both HER2 and androgen receptor were negative. Systemic chemotherapy and comprehensive genomic profiling were discussed; however, the patient was concerned about treatment‐related toxicity, including worsening hearing impairment, and declined systemic therapy because he was asymptomatic at that time.

**Figure 6:**
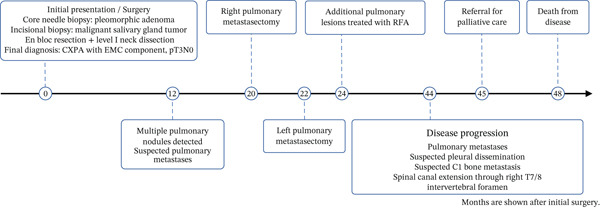
Clinical timeline of the present case. Timeline showing the diagnostic process, surgical treatment, development of pulmonary metastases, subsequent disease progression, referral for palliative care, and death 48 months after the initial surgery. Months are shown after the initial surgery.

At 44 months after surgery, progression of pulmonary metastases, suspected pleural dissemination, suspected C1 bone metastasis, and spinal canal extension through the right T7/8 intervertebral foramen were detected. Because he had no severe neurological symptoms or intractable pain at that time and declined further aggressive treatment, palliative radiotherapy or surgical decompression was not performed. Given the disseminated disease status and the patient′s repeated preference against aggressive treatment, best supportive care was selected. He was referred for palliative care at 45 months after the initial surgery and died of the disease 48 months after surgery. The clinical course is summarized in Figure 6.

## 3. Discussion

The present case illustrates a diagnostic pitfall in salivary gland tumors in which radiological and clinical findings suggested malignancy, whereas the initial core needle biopsy showed only PA‐like features. This discordance is particularly relevant in CXPA because the malignant component may occupy only part of a background PA and may not be represented in a limited biopsy specimen [1, 2]. In the resected specimen, the coexistence of residual PA, biphasic epithelial‐myoepithelial carcinoma, and unequivocal extracapsular invasion supported the diagnosis of CXPA with an EMC component. EMC is characterized histologically by biphasic duct‐like structures composed of inner ductal epithelial cells and outer myoepithelial cells [7]. Although classical EMC often shows outer myoepithelial cells with clear cytoplasm, the outer cells in the present case were predominantly basaloid. Immunohistochemically, ductal epithelial cells are typically positive for epithelial markers, whereas myoepithelial cells are positive for myoepithelial markers such as p63, p40, CK14, SMA, or S‐100 protein [7]. In the present case, the final pathological findings were consistent with CXPA with an EMC component.

The central clinical issue in the present case was the diagnostic discordance between the initial core needle biopsy interpretation of PA and the final diagnosis of invasive CXPA with an EMC component. This discordance was most likely attributable to intratumoral heterogeneity and the basaloid phenotype of the sampled bilayered tubular structures. In CXPA, benign PA components, transitional areas, malignant transformed components, and invasive areas may coexist unevenly within the same tumor. Therefore, a limited biopsy specimen may sample only the benign PA component or PA‐like basaloid tubular areas and fail to capture the malignant area. In the present case, the initial core needle biopsy most likely sampled the PA component and/or a PA‐like basaloid tubular area, whereas the invasive carcinomatous component with EMC differentiation was not adequately represented in the biopsy specimen. This structural relationship between the benign and malignant components explains why a benign or PA‐like biopsy result does not necessarily exclude CXPA when clinical or radiological findings suggest malignancy.

The diagnostic limitations of preoperative biopsy for salivary gland tumors have also been reported, particularly with regard to variable sensitivity and the risk of false‐negative results [8]. Repeat ultrasound‐guided or image‐guided core needle biopsy targeting radiologically suspicious areas, such as irregular margins, areas suggestive of invasion into adjacent tissues, or regions showing strong FDG uptake, could have been considered and might have improved diagnostic accuracy. However, imaging alone may not reliably distinguish the localization of the PA component, EMC component, and invasive carcinomatous component within a heterogeneous CXPA. In the present case, lower‐lip weakness, apparent adhesion to the mandible, irregular imaging features, and increased FDG uptake strongly suggested malignancy despite the provisional biopsy interpretation of PA. We therefore selected incisional biopsy under general anesthesia, with a plan to proceed directly to definitive resection if malignancy was confirmed by intraoperative frozen‐section examination. This strategy allowed diagnostic confirmation and immediate oncologic management. These findings emphasize that, when pathological findings are inconsistent with clinical or radiological suspicion in salivary gland tumors, clinicians should not regard a benign biopsy result as definitive. Instead, the possibility of sampling error should be considered, and additional targeted biopsy, incisional biopsy, or intraoperative frozen‐section diagnosis should be incorporated into clinical decision‐making.

Another important issue was surgical decision‐making regarding the mandible and the marginal mandibular branch of the facial nerve. Although preoperative imaging did not demonstrate definite mandibular invasion, the tumor was in contact with the inferior border of the mandible, and lower‐lip weakness suggested possible involvement of the marginal mandibular branch. Therefore, after malignancy was confirmed intraoperatively, marginal mandibulectomy and en bloc resection of the adjacent soft tissue were performed to secure an adequate oncologic margin. These procedures should be interpreted as margin‐oriented management for a clinically malignant submandibular gland tumor rather than as evidence of radiologically proven bone invasion.

To contextualize the rarity of the present case, we conducted a narrative review of previously published reports between 2005 and 2025. Among the retrieved CXPA reports with available information on the primary anatomical site, the submandibular gland was less frequently reported than the parotid gland and was the second most frequently reported major salivary gland site in this review (Table S1). Among the retrieved cases with available information on the carcinomatous component, EMC was infrequently reported as a carcinomatous component of CXPA (Table S2). These findings represent descriptive observations from the reports identified in the present narrative review and should not be interpreted as epidemiological estimates. Because detailed information on the carcinomatous component was unavailable in many reports, the findings should be interpreted with caution.

Our review of published cases of EMC arising in the submandibular gland also suggested that preoperative diagnosis is difficult, particularly when EMC is associated with CXPA (Table 1). Among the retrieved cases, no case was clearly diagnosed as EMC before surgery [9–20]. Several cases were diagnosed preoperatively as PA or as an unspecified submandibular gland tumor. This descriptive pattern is consistent with the present case and may be explained by the deep anatomical location of the submandibular gland, limited tissue sampling, and the uneven coexistence of PA and malignant EMC components within the tumor. By contrast, EMC arising in more accessible sites such as the nasal cavity, nasopharynx, or tongue may allow more adequate tissue sampling under direct visualization [21–24].

**Table 1:** Reported cases of epithelial‐myoepithelial carcinoma of the submandibular gland, including the present case.

Characteristic	Value
Mean age, years	58.7 (range, 32–80)^a^
Sex	Male, 8/14 (57.1%); female, 4/14 (28.6%); not reported, 2/14 (14.3%)
Maximum tumor diameter (mm), mean	34.1 (range, 18–62)^b^
Clinical impression/diagnosis	Submandibular gland malignant tumor, 3/14 (21.4%); submandibular gland tumor, 6/14 (42.9%); pleomorphic adenoma, 3/14 (21.4%); not reported, 2/14 (14.3%)
Preoperative pathological assessment	Core needle biopsy, 4/14 (28.6%); fine‐needle aspiration, 1/14 (7.1%); not reported, 9/14 (64.3%)
Preoperative histological diagnosis	Pleomorphic adenoma, 4/14 (28.6%); malignant tumor, 2/14 (14.3%); not reported or undetermined, 8/14 (57.1%)
Postoperative histological diagnosis	EMC, 14/14 (100%); EMC arising in CXPA, 5/14 (35.7%)
Treatment	Surgical excision, 14/14 (100%); among surgically treated cases, neck dissection, 5/14 (35.7%); adjuvant radiotherapy, 2/14 (14.3%); chemoradiotherapy, 1/14 (7.1%)
Outcome	Recurrence and/or metastasis, 2/14 (14.3%); disease‐specific death, 2/14 (14.3%); no evidence of recurrence during follow‐up, 12/14 (85.7%)^c^
Follow‐up duration, months	6–48

^a^Calculated from 12 cases because age was not reported in 2 cases.

^b^Calculated from 10 cases because maximum tumor diameter was not reported in 4 cases.

^c^Disease‐specific deaths occurred in the same two patients who developed recurrence and/or metastasis.

In limited biopsy specimens, the basaloid appearance of the outer myoepithelial cells may overlap morphologically with the myoepithelial component of PA and with other basaloid salivary gland tumors, and this overlap probably contributed to the provisional PA interpretation in the present case. PA may also show bilayered tubular structures; however, neoplastic myoepithelial cells in PA are generally more morphologically diverse, and the proliferative activity is usually low. Basal cell adenoma and basal cell adenocarcinoma can also show bilayered tubular structures; stromal characteristics, including prominent hyalinization and S‐100 protein‐positive stromal/fibroblastic cells, together with the presence or absence of infiltrative growth, may be useful in the differential diagnosis. Adenoid cystic carcinoma may also exhibit tubular structures and is often associated with marked perineural invasion, but a cribriform pattern and immunohistochemical positivity for c‐kit and MYB would support that diagnosis [7]. In the resected specimen, the combination of biphasic duct‐like structures, epithelial marker‐positive inner cells, myoepithelial marker‐positive basaloid outer cells, a residual PA component, increased Ki‐67 activity in extracapsular invasive hot spots, and unequivocal extracapsular invasion supported the diagnosis of CXPA with an EMC component.

In the absence of a well‐established nonsurgical curative approach for CXPA or EMC, complete surgical excision remains the cornerstone of management. Prognostic factors for CXPA include the extent of invasion, histological type of the carcinomatous component, tumor size, proportion of the carcinomatous component, Ki‐67 index, cervical lymph node metastasis, and perineural invasion [25, 26]. CXPA is classified as noninvasive, minimally invasive, or invasive according to the extent of extracapsular invasion. Invasion within 1.5 mm is generally regarded as minimally invasive, whereas invasion beyond 1.5 mm is classified as invasive [27]. In the present case, extracapsular invasion exceeded 6 mm and was accompanied by perineural invasion and extension into adjacent muscle. Although the terminology for “widely invasive” CXPA is not uniformly defined across all reports, these findings clearly indicate invasive CXPA with high‐risk pathological features rather than minimally invasive disease. The pT3 classification was based on extraparenchymal extension into adjacent muscle, despite a maximum tumor diameter of approximately 30 mm.

The aggressive clinical course in the present case should be interpreted in light of these established prognostic factors. Although surgical margins were negative and the pathological nodal status was pN0, pulmonary metastases developed 12 months after surgery, followed by pleural dissemination and spinal canal extension. This course suggests that the unfavorable outcome was not primarily related to inadequate initial local control, but rather to the biological aggressiveness of the tumor at the time of initial treatment. Although EMC is generally considered a low‐ to intermediate‐grade malignancy [5, 7], EMC arising in the setting of invasive CXPA may behave more aggressively than conventional EMC. Therefore, in CXPA with an EMC component, prognostic assessment should be based not only on the histological label of EMC but also on the invasive status of CXPA and the presence of high‐risk pathological features. In addition, Ki‐67 expression showed intratumoral heterogeneity. Although the overall Ki‐67 estimate was approximately 10%, the Ki‐67 index was 27% in hot spots of the extracapsular invasive component. Ki‐67 index has been reported as a prognostic factor in CXPA [25, 26]; therefore, the hotspot value may better reflect the proliferative potential of the invasive component than the overall estimate alone. Direct numerical comparison with high‐grade CXPA cases is limited by the rarity and histological heterogeneity of CXPA with an EMC component, but the presence of a 27% invasive hotspot helps explain the apparent discrepancy between the overall 10% index and the lethal clinical course. In the present case, the aggressive clinical course was considered to be associated with both elevated hotspot Ki‐67 activity and established high‐risk features, including extracapsular invasion, perineural invasion, and muscle involvement.

The postoperative management of this case also requires careful interpretation. Although complete resection with negative margins was achieved, the presence of high‐risk features, including perineural invasion and muscle involvement, would generally warrant consideration of postoperative radiotherapy. Postoperative radiotherapy has been reported to improve local control in CXPA [28]. In this case, adjuvant radiotherapy was considered but was not administered because the patient preferred to avoid treatment‐related toxicity after discussion of the potential risks and benefits. However, given the presence of multiple high‐risk pathological features, the oncologic appropriateness of omitting postoperative radiotherapy cannot be determined from this single case and remains an open question.

For recurrent or metastatic CXPA with an EMC component, treatment selection is difficult because no standard systemic regimen has been established for this specific setting. Previous reports have described chemotherapy regimens selected according to the carcinomatous component, including platinum‐based chemotherapy or taxane‐containing regimens [29, 30]. Trastuzumab‐based therapy has also been reported in HER2‐positive CXPA [31]. In addition, chemotherapy has been reported for metastatic EMC, although its efficacy remains unclear [32]. In the present case, HER2 and androgen receptor were evaluated after the development of pulmonary metastases, not because they are characteristic markers of EMC, but because CXPA can show diverse carcinomatous differentiation and these markers may influence systemic treatment options in advanced salivary gland carcinoma. However, both HER2 and androgen receptor were negative; therefore, HER2‐targeted therapy and androgen blockade were not indicated [33, 34].

After pulmonary metastases became evident, local treatment for metastatic lesions, including pulmonary metastasectomy and radiofrequency ablation, was selected because the lesions were limited and systemic therapy had no established standard regimen for this histological subtype. Later in the disease course, systemic chemotherapy and comprehensive genomic profiling were discussed; however, the patient repeatedly declined systemic therapy because of concerns about treatment‐related toxicity, including worsening hearing impairment. Given the subsequent progression of pulmonary metastases, pleural dissemination, suspected bone metastasis, and spinal canal extension, best supportive care was ultimately selected. This clinical course underscores the difficulty of selecting systemic therapy for rare salivary gland carcinomas and the importance of incorporating patient preference into treatment decision‐making.

Several limitations should be acknowledged. First, molecular analyses, including PLAG1 and HMGA2 rearrangement testing and HRAS mutation analysis, were not performed. HRAS mutations have been reported in EMC, although their frequency may be lower in EMC arising from PA or CXPA [35–37]. Consequently, the PA origin and the molecular characteristics of the EMC component could not be independently corroborated at the molecular level. Although the diagnosis in the present case was supported by the coexistence of a residual PA component, biphasic duct‐like carcinomatous structures, immunophenotypic evidence of epithelial and myoepithelial differentiation, and unequivocal extracapsular invasion, molecular analyses could have provided additional support for the diagnosis and tumor characterization. Second, this is a single case report, and generalization is limited. Third, the literature review was narrative and not systematic; therefore, publication, selection, language, and database biases cannot be excluded. The aggregate data should be regarded as descriptive summaries of the retrieved reports and not as epidemiological estimates. Fourth, because the initial biopsy did not target a radiologically suspicious invasive area under image guidance, a more targeted biopsy strategy might have identified the malignant component preoperatively.

In conclusion, this case highlights the diagnostic complexity of CXPA with EMC differentiation in the submandibular gland. A benign core needle biopsy diagnosis does not necessarily exclude CXPA when clinical and radiological findings suggest malignancy, because benign PA and malignant components may coexist unevenly within the same tumor. When biopsy findings are discordant with neurological symptoms or imaging findings suggestive of malignancy, clinicians should consider sampling error and plan diagnostic and therapeutic strategies that accommodate the possibility of malignant transformation. Although the present case cannot establish the optimal postoperative management strategy, invasive CXPA with high‐risk pathological features requires close follow‐up, and the role of adjuvant therapy should be carefully considered after complete resection.

## Author Contributions

Tomohiro Yasuhara contributed to investigation, data curation, and writing of the original draft. Masanori Masui contributed to conceptualization, investigation, data curation, formal analysis, visualization, project administration, and writing—review and editing. Yuki Kunisada, Hiroaki Takakura, Eiji Iwata, Koichi Kadoya, Koki Umemori, and Norie Yoshioka contributed to investigation and writing—review and editing. Keisuke Nakano contributed to pathological investigation, data curation, visualization, and writing—review and editing. Soichiro Ibaragi contributed to supervision, project administration, and writing—review and editing.

## Funding

No funding was received for this manuscript.

## Disclosure

All authors have read and approved the final version of the manuscript.

## Ethics Statement

Ethical approval was not required for this study according to our institutional policy because this report describes a single anonymized case and written informed consent for publication was obtained from the patient. The report was prepared in accordance with the principles of the Declaration of Helsinki.

## Consent

Written informed consent was obtained from the patient for publication of this case report and the accompanying images.

## Conflicts of Interest

The authors declare no conflicts of interest.

## Supporting information


**Supporting Information** Additional supporting information can be found online in the Supporting Information section. Table S1: Primary sites of CXPA cases extracted from 537 eligible publications in the present narrative review, 2005–2025. Table S2: Carcinomatous components among CXPA cases with available histological subtype information extracted from eligible publications in the present narrative review, 2005–2025.

## Data Availability

All data related to the present case are included in this published article.
