# Supplementary material for: Carcinoma ex Pleomorphic Adenoma With an Epithelial‐Myoepithelial Carcinoma Component in the Submandibular Gland: A Case Report
Source: Case Rep Dent. 2026 Jul 17;2026:7698933. doi: 10.1155/crid/7698933 (PMC13377794; doi:10.1155/crid/7698933)
Supplement: Supplementary file 1 — Supporting Information Additional supporting information can be found online in the Supporting Information section. Table S1: Primary sites of CXPA cases extracted from 537 eligible publications in the present narrative review, 2005–2025. Table S2: Carcinomatous components among CXPA cases with available histological subtype information extracted from eligible publications in the present narrative review, 2005–2025. [file CRID-2026-7698933-s001.docx]

**Supplementary Table 1.**

| Primary site | n | % |
| --- | --- | --- |
| Parotid gland | 3942 | 78.26 |
| Submandibular gland | 618 | 12.27 |
| Minor salivary glands | 157 | 3.12 |
| Lacrimal gland | 57 | 1.13 |
| Sublingual gland | 22 | 0.44 |
| Other sites (e.g., nasal cavity, pharynx, maxillary sinus, lung, and breast) | 241 | 4.78 |
| Total | 5037 | 100.00 |
| Data were extracted by the authors from 537 eligible publications identified in the present narrative review. Cases were included when information on the primary anatomical site of CXPA was available. The data were not derived from an epidemiological database or a single previous meta-analysis. | | |

**Supplementary Table 2.**

| Carcinomatous component | n | % |
| --- | --- | --- |
| Salivary duct carcinoma | 655 | 43.38 |
| Myoepithelial carcinoma | 331 | 21.92 |
| Adenocarcinoma, NOS | 144 | 9.54 |
| Epithelial-myoepithelial carcinoma | 95 | 6.29 |
| Adenoid cystic carcinoma | 54 | 3.58 |
| Adenocarcinoma | 52 | 3.44 |
| Undifferentiated carcinoma | 47 | 3.11 |
| Mucoepidermoid carcinoma | 43 | 2.85 |
| Squamous cell carcinoma | 28 | 1.85 |
| Ductal carcinoma | 18 | 1.19 |
| Basal cell adenocarcinoma | 12 | 0.79 |
| Carcinosarcoma | 11 | 0.73 |
| Carcinoma in situ | 6 | 0.40 |
| Oncocytic carcinoma | 4 | 0.26 |
| Secretory carcinoma | 3 | 0.20 |
| Poorly differentiated carcinoma | 3 | 0.20 |
| Acinic cell carcinoma | 2 | 0.13 |
| Clear cell carcinoma | 1 | 0.07 |
| B-cell chronic lymphocytic leukemia/lymphoma | 1 | 0.07 |
| Total | 1510 | 100.00 |
| Data were extracted by the authors from the same 537 eligible publications used for Supplementary table 1. This table includes only cases in which the histological subtype of the carcinomatous component was explicitly described. Cases without sufficient information regarding the carcinomatous component were excluded from this table; therefore, the denominator differs from that in Supplementary table 1. The proportions shown in this table represent the distribution among cases with available histological subtype information, not among all CXPA cases.  Cases reported simply as “adenocarcinoma” were not reclassified as “adenocarcinoma, NOS” and were analyzed separately, because the original descriptions lacked sufficient histopathological detail for accurate subclassification. | | |
